# Motivation as an important criterion for graduation among medical students admitted from the waiting list

**DOI:** 10.3205/zma001214

**Published:** 2019-02-15

**Authors:** Carolin Verena Herbst, Brigitte Müller-Hilke

**Affiliations:** 1 Universitätsmedizin Rostock, Institut für Immunologie, Rostock, Germany

**Keywords:** medical school admission, NEO-Five-Factor-Inventory, drop-out, motivation

## Abstract

**Aim: **Graduation rates among medical students who have been admitted to medical school from the waiting list quota are significantly lower than those for medical students who are directly admitted on the basis of their competitive secondary school academic record or through the universities’ selection process. The aim of this study was to identify risk factors that can predict a longer length of study or dropping out and potential resilience factors predictive of timely and successful graduation.

**Method: **First, students admitted from the waiting list were asked in qualitative interviews at the beginning of their studies about risk factors connected with dropping out. These students were then followed until completion of the first state medical exam, or at least until the end of their fourth semester.

In parallel, personality traits were measured using the NEO Five-Factor Inventory according to Costa and McCrae (NEO-FFI). Successful study was defined as a length of study time lasting four semesters before taking the first section of the state medical exam (Physikum). Serving as indicators for students at risk were a prolonged period of study and dropping out before taking the first state medical examination. Finally, the factors associated with successful study were identified.

**Results: **Students from the waiting list who displayed a stronger than average conscientiousness in their personality and stated being underchallenged in their prior (medicine-related) occupation as the motivation for studying medicine were significantly more often successful than students from the waiting list who displayed a less pronounced conscientious personality and named dissatisfaction with their previous occupation as their motivation to pursue medical study. In addition, successful students were often distinguished by ambition and reported placing high academic demands on themselves. Early failures on exams were found to be predictive of an uncertain course of study at Rostock Medical School.

**Conclusions:** The reason for studying medicine and an ambitious personality appear to be basic predictors of study success and could therefore be considered not only as a selection criterion for admission, but also monitored during the course of study as a predictive marker for prolonged study or drop-out. Regardless how students are selected for admission, medical schools should take a closer look at the academic performance of the enrolled students to identify at-risk students who are failing exams early in the course of study and to adequately advise them on course scheduling, motivation and exam preparation.

## Zusammenfassung

**Zielsetzung: **Die Erfolgsquoten von Medizinstudierenden mit Zulassung über die Wartezeitquote sind deutlich geringer als die derjenigen Medizinstudierenden, welche über die Abiturbestenquote oder das AdH (Auswahlverfahren der Hochschulen) zugelassen werden. Ziel dieser Studie war es, sowohl Risikofaktoren, die eine Verzögerung der Studiendauer oder einen Studienabbruch frühzeitig anzeigen können, als auch mögliche Resilienzfaktoren, welche prädiktiv für einen zeitnahen, erfolgreichen Studienabschluss stehen, zu identifizieren.

**Methodik: **Zunächst wurden Wartezeitstudierende zu Beginn ihres Studiums in qualitativen Einzelinterviews nach Risikofaktoren zum Studienabbruch befragt und dann bis zum Abschluss des ersten Staatsexamens – oder aber mindestens bis zum Ende ihres vierten Semesters begleitet.

Parallel wurden Merkmale zum Persönlichkeitsinventar mithilfe des NEO-Fünf-Faktoren-Inventars nach Costa und McCrae (NEO-FFI) erhoben. Als Kategorie für erfolgreiche Studierende galt eine Studiendauer von vier Semestern bis zum Physikum (Regelstudienzeit), für Risikostudierende galt sowohl eine verlängerte Studiendauer, als auch der Studienabbruch vor Erreichen des ersten Staatsexamens. Abschließend wurde erfasst, welche Faktoren mit einem erfolgreichen Studienverlauf in Zusammenhang stehen.

**Ergebnisse: **Wartezeitstudierende, die sich durch ein überdurchschnittlich gewissenhaftes Persönlichkeitsprofil auszeichnen und als Motivation zum Medizinstudium Unterforderung im vorherigen (medizinnahen) Ausbildungsberuf angeben, waren signifikant häufiger erfolgreich als Wartezeitstudierende, die sich durch ein weniger gewissenhaftes Persönlichkeitsprofil auszeichnen und als Motivationsgrund Unzufriedenheit im vorherigen Ausbildungsberuf benannten. Zudem bezeichneten sich erfolgreiche Studierende gehäuft als ehrgeizig und gaben einen hohen persönlichen Leistungsanspruch an. Frühe Prüfungsmisserfolge erwiesen sich im Rahmen der lokalen Prüfungsanforderungen der Universitätsmedizin Rostock zudem als prädiktiv für einen unsicheren Studienverlauf.

**Schlussfolgerungen: **Motivation zum Medizinstudium und ein ehrgeiziges Persönlichkeitsprofil scheinen wesentliche Erfolgsprädiktoren für den Studienverlauf zu sein und können daher sowohl als Auswahlkriterium in Betracht, als auch während des Studiums als prädiktiver Marker für eine Verzögerung der Studiendauer oder einen Studienabbruch herangezogen werden. Unabhängig von der Vorauswahl der Studierendenschaft sollten sich die jeweiligen Fakultäten jedoch auch verstärkt mit lokalen Leistungsnachweisen auseinandersetzen, um hier Risikostudierende mit frühen Prüfungsmisserfolgen zu identifizieren und bezüglich Studienplanung, Motivation und Prüfungsvorbereitung adäquat zu beraten.

## Introduction

### The waiting list as a special option for admission in Germany

Medical school applicants are admitted to medical school in Germany through a complex, multi-step process which allots the absolute number of available spaces according to specific quotas [https://zv.hochschulstart.de/index.php?id=281 last verified on 19 August 2018]. Twenty percent of spaces are assigned centrally to the applicants with the highest scores on the school-leaving exam (academic merit-based admission) and 20% of spaces to applicants who have been on the waiting list the longest (waiting list quota). The remaining 60% are allotted through the universities’ selection process which is carried out according to criteria that are not uniform nationally and in which the score on the school-leaving exam is usually the pivotal admission criterion [https://zv.hochschulstart.de/index.php?id=281 last verified on 19 August 2018]. As this selection process and in particular the waiting list quota have been heavily criticized for many years, the Federal Constitutional Court declared the current admission procedures for partially unconstitutional in December 2017 and instructed lawmakers to redesign the process by the end of 2019 [1], [http://www.bverfg.de/e/ls20171219_1bvl000314.html last verified on 19 August 2018]. Based on this court judgment, the Cultural Ministers’ Conference (Kultusministerkonferenz) decided to abolish the waiting list quota while still allowing a transitional phase for the current applicants on the waiting list during which the time spent on the list will still count. Simultaneously, two new criteria, in addition to the score on the school-leaving exam, will be applied in the future by the universities in their selection process [2]. A recurring topic of debate is the presence of relevant and completed occupational training.

In the 2015/16 winter semester the waiting period in Germany was already 14 semesters and thus longer than the intended full-time medical degree program of six years and three months [https://zv.hochschulstart.de/fileadmin/media/zv/nc/wise2015_16/nc_zv_ws15.pdf last verified on 19 August 2018]. If the applicants seeking to enter medical school under this quota are admitted after seven years of waiting, they not only increase the diversity of the student body, but also very likely bring a range of resilience factors with them that are potentially predictive of study success. Among these resilience factors are a desire for further education, a realistic view of the medical profession, a solid personality and a strong motivation to study medicine [3], [4].

#### Students admitted from the waiting list drop out more frequently

Despite this, only few students from the waiting list complete their medical degrees within the time intended and around 25% of the students from the waiting list drop out [5], [6]. Compared to a drop-out rate of about 5% for all medical students, this is a high percentage and is not only experienced as a personal failure by those affected [7], but also comes with economic losses for the medical schools. Academic struggles and failed exams are being discussed as significant reasons to discontinue medical studies [6]. Other relevant reasons for dropping out are primarily low motivation and problems with financing university study [7].

These reasons could apply to the student drop-outs who were on the waiting list: in addition to lower school-leaving grades, they also had a long pause between graduating from secondary school and beginning their medical studies, which makes entering higher education and adjusting to the demands difficult [6], [7]. They are also on average seven years older than their fellow students who were admitted directly after completing secondary school and they are often farther along in terms of getting married and starting a family [8]. If they already have children, then valuable time is missing for exam preparation and attending lectures, something that can negatively affect the pre-clinical study phase, in particular [9]. Also, students admitted from the waiting list experience a substantial decline in living standard in that they give up their financial independence to finance their studies while foregoing job-related earned income and becoming dependent on public or private financial aid. Finally, even poor health, difficult or inadequate study conditions or a change of career can also be reasons for dropping out [7].

#### Reasons for successful academic study

However, according to our knowledge, there has not been sufficient investigation as to whether there are predictors of success which successful students from the waiting lists bring with them or what these predictors might be [6], [10]. We undertook to investigate these questions in this study. Our aim was to identify factors with which it is possible to differentiate between students from the waiting list who will successfully complete their studies within the time intended and at-risk students who will take longer to complete their studies or even drop out without graduating [3], [10].

#### Drop-out rate and attrition rate

The relevant literature differentiates between a drop-out rate for students who leave the university without graduating and do not go on to study another subject and an attrition rate which measures the fluctuation caused by students changing courses of study or transferring to other universities [11]. Since these personal life decisions cannot always be understood in the context of this study, we will speak of “discontinuation” when students terminate their studies at the University of Rostock Medical School without stating concrete plans to study medicine at another university.

Previous studies have shown that discontinuing medical study occurs most often in the preclinical semesters [6], [7]. Research on the reasons for discontinuation is made difficult by the fact that students who terminate their studies often disenroll from the particular medical school without citing reasons. For this reason, we chose to pursue a prospective study in which students from the waiting list were surveyed early in their studies in structured interviews about their personal risk factors concerning a discontinuation of their studies and their resilience factors. These students were then continually followed until the first state medical exam. In doing this, we wanted to learn the reasons for discontinuing university study as early as possible from the students and, based on the required length of time leading up to the first state medical exam, differentiate between students who were successful in their study from those who were facing uncertainties about completing. Ideally, these differences would enable us to identify early on the students struggling to successfully complete their medical studies so that they could be offered support and advice to prevent delayed graduation or discontinuation of medical study.

## Method

### Sample

A total of n= 38 students who were admitted from the waiting list participated in this study. Recruitment was carried out in the 2015/16 and 2016/17 winter semesters in required lecture courses normally offered to third-semester students. Since these lectures were not only for third-semester students, but also attended by students at a more advanced semester level, our cohort consisted of representatives from a total of four semester levels. Overall, 2% (1/38) of the participating students had enrolled in 2012/13, 26% (10/38) in 2013/14, 46% (16/35) in 2014/15, and 36% (11/31) in 2015/16.

Table 1 (Tab. 1) summarizes the demographic data of the participants: 53% were female, mean age was 28 years and 13% were already parents at the time of the survey.

#### Ethics

All participants were briefed about the study’s content and aims and gave their written consent to participate in the collection of data. This study was reviewed by the local ethics commission and no objections were made (file no.: A 2015-0132).

#### Survey

Using the risk factors for discontinuing university study in Germany described by Heublein et al. [7], interview guidelines were developed to ask participants about their personal risk factors and potential resilience factors for successful study. The survey was divided into eight sections according to topic:

1. motivation for university study;
2. difficulties with the academic demands or the amount of material;
3. exam failures;
4. difficulties financing university study;
5. inadequate study conditions indicated by a lack of integration into the peer group or lack of flexibility in course scheduling due to strict requirements imposed by the medical school;
6. poor health;
7. personal/family reasons;
8. discontinuation of studies due to a change of career.

In addition, the participants’ personality traits were measured using the NEO Five-Factor Inventory (NEO-FFI), and age-normed T values were calculated [12].

#### Approach

This study is a prospective follow-up study. The interview guidelines were first tested on two students in the clinical study phase to determine the comprehensibility of the questions and the unfolding of a logical, coherent discussion; participants were then recruited on a volunteer basis. Individual interviews were scheduled with each participant; an average of 25 minutes was needed to ask the questions in the guideline. All interviews were held by the same person (CH), recorded and then transcribed verbatim. Up until they successfully passed the first state exam (Physikum), the participants were contacted regularly, at least once per semester to remain informed about any changes in plans, study progress and the possibility of discontinuation. Data collection was complete when a participant passed the first state exam or if there was no response to multiple attempts to make contact, whereby in these cases the Dean of Studies was contacted to find out if the participant had successfully passed the first state exam during the observational period. Discontinuing participants were invited to a final interview, which two of four participants did agree to.

#### Analysis

The primary coding of the interviews was done independently by both authors using the software MAXQDA (version 12.3.5) before being qualitatively analyzed with quantitative elements [13]. Using the arguments cited, categories were created to summarize the statements made by the participants. Then the successful students who had completed the preclinical study phase within the intended length of time were compared with the at-risk students who had taken their first state exam after a delay or who had discontinued medical study prior to attaining the first state exam in terms of the statements they made and their personality traits. Fisher’s exact test was applied to calculate the level of significance of the differences between successful and at-risk students in the identification of arguments. An unpaired t-test was used to compare the personality profiles.

## Results

### Thirteen of 38 students admitted from the waiting list had taken their first state exam within the normal period of time

At study conclusion, 34% of the students had successfully passed the first state exam within the standard timeframe of four semesters. More than four semesters were needed by 50% to complete the preclinical study phase; 11% had discontinued medical study prior to the first state exam and 5% had transferred to another university after the standard period of study had been exceeded.

#### The vast majority of the students admitted from the waiting list had completed educational programs in a healthcare occupation

Overall, 95% had already attained professional qualifications in an occupation closely related to medicine and had already gathered professional experience. A total of 42% were trained (pediatric) nurses, 18% physiotherapists, 11% operating department technicians (*OTA*), 8% paramedics, 5% speech therapists, 5% radiology technicians (*MTRA*), and yet another 5% had completed other training closely related to healthcare. Only 5% of the study participants had not worked in the healthcare sector prior to taking up the study of medicine (see table 1 (Tab. 1)).

#### Feeling unchallenged in the previous occupation appears to be a stronger predictor of success than dissatisfaction

Successful medical study was measured based on the number of semesters required for the preclinical study phase. Since all participants would have had the opportunity to take the first state exam within the standard period of time, we divided the participants into two groups: the 13 successful students who passed the first state exam within the intended time period of four semesters and the 25 at-risk candidates who needed longer than four semesters before taking the first state exam and thus exceeded the normal period of study or discontinued their studies. The transcribed interviews were then analyzed for differences in the statements made by these two groups.

In the individual interviews the participants were asked to share their personal motivation for studying medicine. With a waiting period of seven years before entering medical school, the presence of a very high level of motivation must be assumed, which could be further reinforced by a realistic and detailed idea of the medical profession and what the practice of medicine entails. The individual statements were coded and summarized thematically in subcategories; it was possible for participants to name different categories. The following seven response categories emerged in connection with the motivation for studying:

1. enthusiasm for medicine came from personal experiences in the prior occupation;
2. late decision to study medicine due to a need to mature first before selecting a career;
3. positive encouragement from others;
4. dissatisfaction with the previous occupation;
5. lack of meaningful challenge in previous occupation;
6. desire to study medicine could not be immediately realized due to an uncompetitive academic record and the admission quotas placed on medical schools; and
7. a relative or friend is a member of the medical profession.

**Figure 1:**
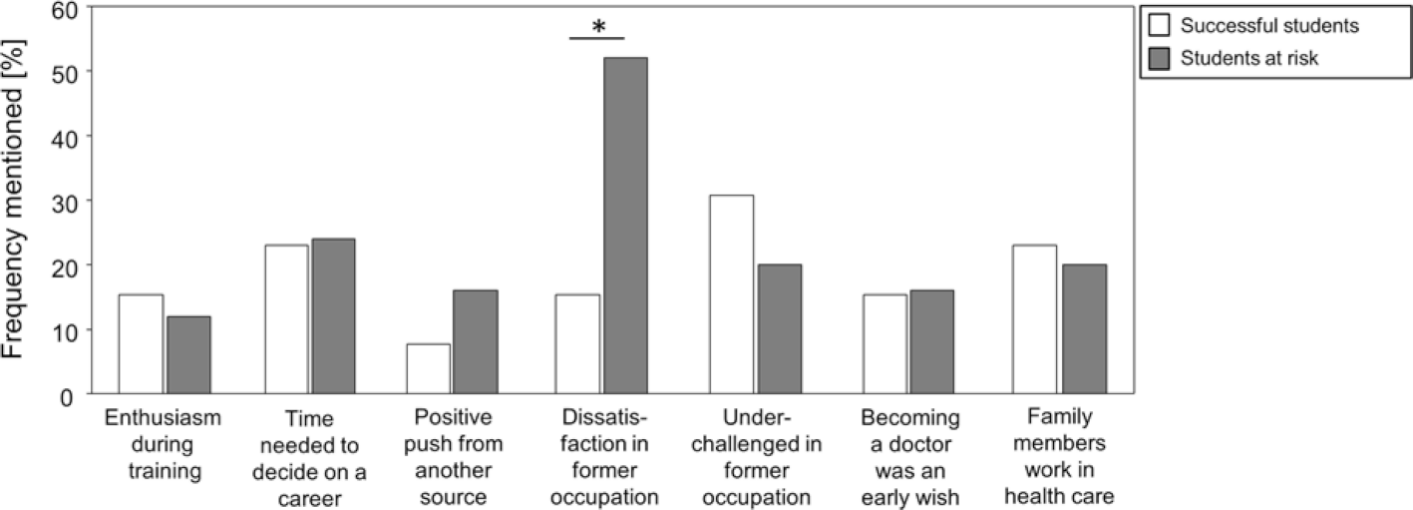
The bar graph shows the frequency of times the different reasons were cited for pursing the study of medicine. The light-colored bars represent the successful students who passed the first state exam (*Physikum*) within the standard number of semesters. The darker bars depict the at-risk students who either studied longer than the standard number of semesters before passing the first state exam or discontinued their medical studies. The asterisk indicates the statistically significant difference between successful and at-risk students in the identification of dissatisfaction with the former job. Fisher’s exact test was performed for the calculation.

Figure 1 (Fig. 1) shows that all of the categories were mentioned by successful and at-risk students. However, there were significant differences between the two groups when stating the reason for studying medicine: at-risk students more frequently cited dissatisfaction with their previous occupation as the impetus for pursuing medical study. We included negative statements that expressed a wish for professional change in the category of occupational dissatisfaction; this was often accompanied by the feeling of being better able to practice medicine than the physicians with whom the students had formerly worked. As an example, study participant S28 stated: *“I did not want to be an assistant anymore, a medical assistant, that was ... the main reason, really. Always standing in the background and watching what exciting things the doctors were doing.”* And as study participant S19 stated: *“And that strengthened my decision even more, so to speak ... because I had come to know doctors and thought to myself: it just isn’t possible. I could certainly do that just as well because the doctors simply weren’t quick to recognize the most important things or in part because they related to others in such a way that left me aghast.”*

Experiencing a lack of occupational challenge, in contrast, was mentioned more often by the successful students than the at-risk students. This category included positive expressions of a desire to know and learn more and to gain a more in-depth understanding of medicine. As an example, study participant S05 remarked: *“Before I began studying medicine, I was a pediatric nurse and I just wanted to learn more; nursing training didn’t go far enough and I really wanted to keep learning.”*

A further example is found in the statement by study participant S31:* “I find that medicine is a bit like a puzzle. At first we don’t know very much about the body and then somehow we learn more and can make more and more connections. In nursing it is such that you learn puzzle pieces A and C with the consequence that you can only work superficially because the connection between the pieces was never explained. I just wanted to know. And the only way to do that was through studying medicine; only the study of medicine can give me that.”*

#### Successful students describe themselves as being very ambitious; at-risk students distance themselves from concerns about academic performance

**Figure 2:**
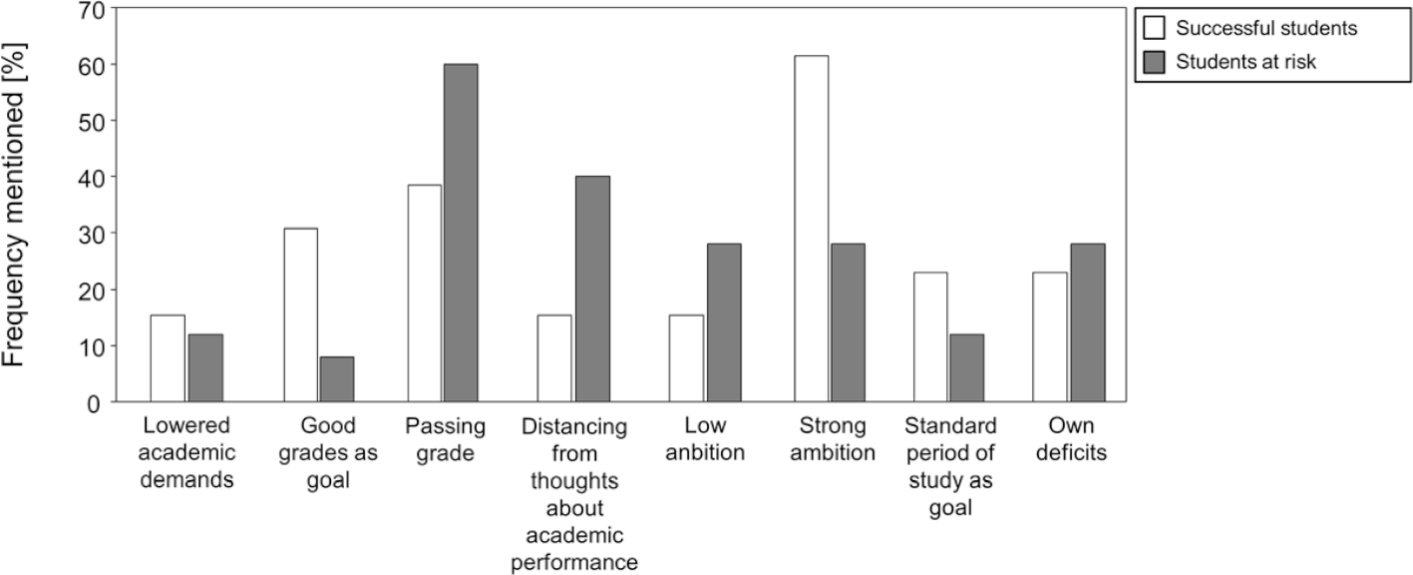
The bar graph shows the frequency of times the ambition categories were cited as reasons for studying medicine. The light-colored bars represent the successful students who passed the first state exam (*Physikum)* within the standard number of semesters. The darker bars depict the at-risk students who either studied longer than the standard number of semester before passing the first state exam or discontinued their medical studies.

To ascertain if successful completion of the preclinical study phase or if an at-risk course of study can be predicted by an early feeling of being over-challenged, we asked the study participants about the academic demands they placed on themselves and their own sense of ambition. Here, too, general categories emerged in which we were able to compile the most frequent responses (see figure 2 (Fig. 2)). These were:

1. a lowering of personal demands since beginning study,
2. good grades as a goal,
3. achieving the passing score as a goal,
4. a distancing from concerns about academic performance,
5. low personal ambition, vi) strong personal ambition,
6. graduating on schedule as a goal, and
7. perceptions of personal inadequacy.

Both successful and at-risk students stated that they had lowered the demands they placed on themselves as a result of the sheer amount of material to be learned and had experienced their own shortcomings. In addition, the successful students more often described themselves as being very ambitious and cited good grades and graduating on time more frequently as goals. In contrast, the at-risk students who prolonged or discontinued their studies more often described themselves as not being ambitious and stating that just passing all the preliminary exams was fully sufficient. For instance, study participant S22 reported, *“The demands I place on myself are such that I hope to get through my medical studies, meaning that a score of four is enough; passing is important to me, but the grade less so.”*

Never the less, some at-risk students distance themselves from any thoughts about their academic performance. Study participant S11 stated, *“Later I will need to be able to handle the responsibility of having a person seated in front of me who does not know what is wrong and who I want to treat adequately. What happens between now and then, to be honest, doesn’t really matter to me.”*

#### Early exam failures indicate problems already at the beginning of study

The preclinical study phase in the standard medical curriculum covers the natural sciences and basic medical subjects for which at the University of Rostock Medical School a total of 16 formal certificates, each documenting the completion of multiple academic requirements, must be successfully obtained for admission to sit for the first state exam. Due to the tight schedule, it is almost only possible to take the first state exam at the intended time if all the required course exams have been passed on the first attempt or, at the latest, on the second. At nearly all of the times they were asked, the 13 successful students in the cohort had successfully passed these course exams on the first attempt. What is remarkable for us, however, is that the at-risk students who were taking longer were already failing exams in the early phases. During the first two semesters the students attended introductory courses on the natural sciences and basic human anatomy. Standing out here are two anatomy exams of which the one taken in the first semester on the general musculoskeletal system was passed by over 93% of the successful students but only by 76% of the at-risk students. In the second semester a second anatomy exam (organ systems) is administered at the University of Rostock Medical School, the failure of which on the first attempt correlates with taking longer to complete medical studies. Due to the provisions for accommodating difficulties, exam failures rarely result in disenrollment since the number of repeat attempts to pass is not limited to three, but rather decided on a case basis by a commission.

Indeed, none of the four students in the cohort who discontinued medical study were disenrolled by the university, rather the two students who agreed to be interviewed again stated that the final decision to discontinue was based on a combination of a lack of motivation to prepare for exams and too much anxiety regarding exam failure.

#### Financing university study played a secondary role for students admitted from the waiting list

For students who had worked professionally for many years, starting medical school meant a financial adjustment, but one that appeared to play a secondary role. The majority of students indicated that they supported themselves through financial aid (Bafög) granted independently of their parents’ financial status and through additional support from parents/partner and/or a part-time job, very often in their previous profession. Even when 79% of the respondents stated that their financial situation had deteriorated compared to the time prior to beginning medical study, the majority did not feel this to be a source of stress. Students had been aware of the financial constraints in advance and were able to adjust to the new situation; on the other hand, there was no longer the opportunity to spend a lot of money given the new daily schedule. The students with children mostly found themselves in stable relationships in which the employed partner helped to stabilize the financial situation. Contrary to the results seen by Heublein et al. [7], the students admitted from the waiting list did not discontinue the study of medicine because of financial difficulties.

#### Inadequacies regarding the study program, poor health or personal/familial reasons played a secondary role for students admitted from the waiting list

Despite much criticism of the conditions imposed by study program, no student in our study discontinued university study due to the absence of adequate conditions for study. There was, however, the impression that the students who had worked in healthcare prior to beginning their medical studies and had a clear understanding of the realities of medical practice may have been more motivated in a practice-based curriculum. As an example, study participant S10 stated, *“I started with an entirely different perspective on medicine and that certainly makes itself felt in that I have a strong focus on relevance to practice and can’t really do much with the material as it is presented now. And now to approach medicine again in a completely different way, first of all, I have to figure out for myself how to do this.”*

Likewise, no student in our cohort discontinued their studies for the reason of poor health. The existence of personal or family-related reasons can only be speculated about in retrospect. Both successful and at-risk students in our cohort cited stress factors such as the demands of their children which limited time to prepare for exams, separations or other major events. Based on the small number of cases here it is impossible to establish any correlation with study success.

#### Study discontinuation as a result of changing careers was rare in students admitted from the waiting list

Most of the students interviewed by us saw their former occupation as a contingency plan in case they failed medical school. Still, only very few wanted to return to their former occupation and derived motivation from this to successfully finish their medical studies. In the case of failure, there was a predominant wish to remain in the healthcare sector. Among those who discontinued, we documented one case of a switch to a dual study program in which the student returned to the former occupation, one case of a discontinuing student who switched to a completely different field for family reasons, and in yet another case the future career path remained unknown.

#### Successful students more often have a conscientious personality

**Figure 3:**
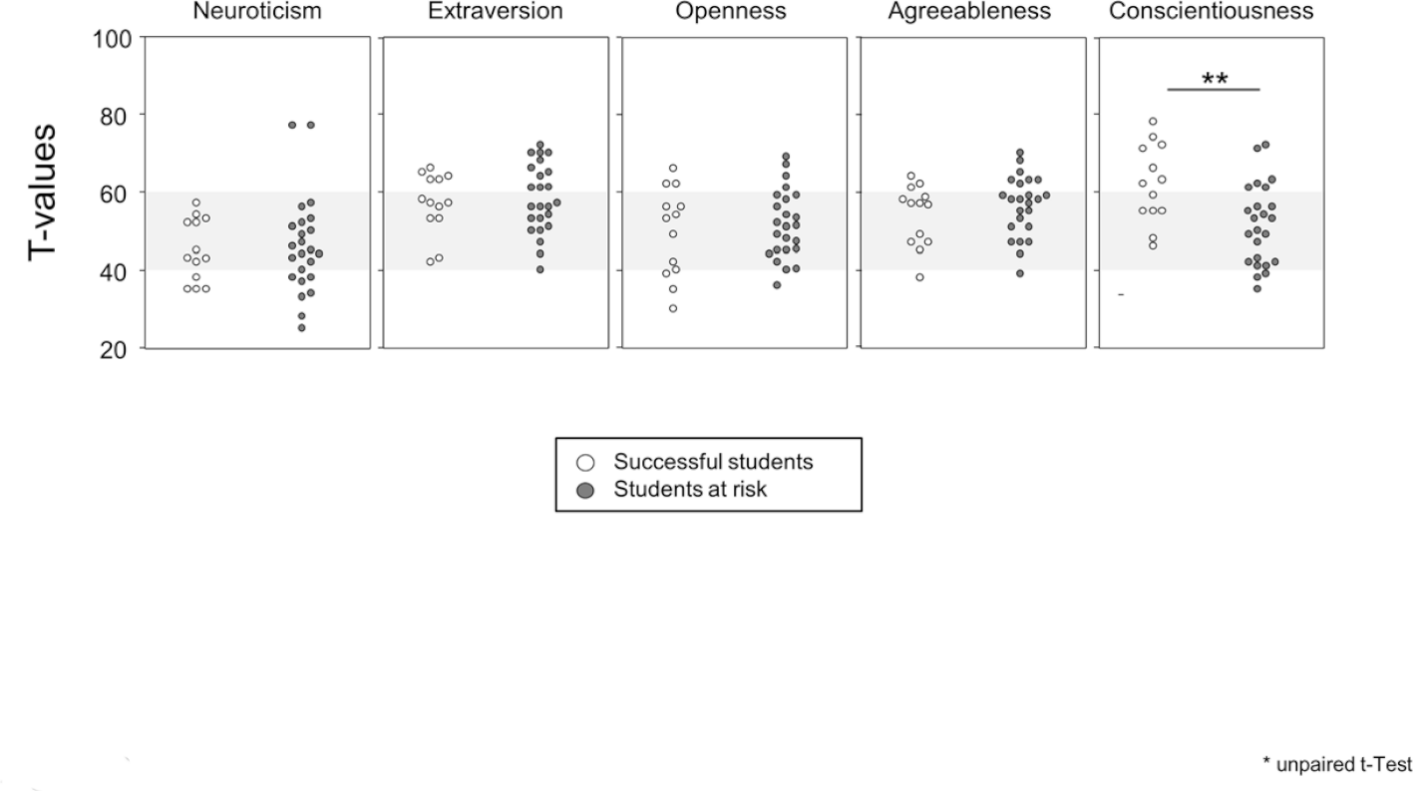
The scatter diagrams show the results of the NEO-FFI for the different personalities as T values. The mean values ± two standard deviations are shown by gray bars. Each dot represents a student: the lighter dots indicate successful students, the darker ones stand for at-risk students. Unpaired T tests were carried out for the calculations.

Using the NEO-FFI, a personality inventory was generated for all of the participants and the results for students successfully completing studies within the standard period of time were compared with those for the students at risk of discontinuing their studies. Figure 3 (Fig. 3) shows that the two groups do not differ from each other in terms of neuroticism, extraversion, openness to experiences or agreeableness. However, the successful students did stand out with an average T value of 61 for conscientiousness and, as a result, were not only more than two standard deviations above the age norm, but also were significantly more conscientious than their fellow students who were at risk of discontinuing.

## Discussion

In this prospective study we have surveyed medical students admitted from the waiting list during their third semester concerning their personal risk factors for discontinuing their medical studies, any concrete thoughts about discontinuing, and possible resilience factors connected with study success [10]. The study participants were then contacted regularly until they passed the first state exam or study end – at least over the course of two semesters – to find out about their academic progress or any decisions to discontinue. The study participants were recruited from three academic cohorts based on year of study and with 26% correspond to a good fourth of all students admitted from the waiting list during the entire time period. The fact that in our cohort 66% of the students admitted from the waiting list were not able to finish within the standard period of time, while an earlier study observed this as being 40%, may be due to our recruiting practice which included students who were repeating [5].

Our grouping of the study participants into successful ones who took the first state exam within the intended time period and at-risk students who needed longer or discontinued their medical studies prior to qualifying to take the first state exam thus displaying a vulnerable course of study may seem rigid, but it appeared sensible given the small number of cases [14], [15]. This division enabled the identification of significant differences between the two groups. It could be seen that successful students possessed a higher than average level of conscientiousness combined with a positively articulated wish to increase their knowledge and further their education and strong personal ambition.

Earlier studies have indeed shown that conscientiousness as a personality trait predicts study success in the preclinical study phase [16], [17]. In that our findings now corroborate these studies, they substantiate the comparability of our cohorts with the study bodies at medical schools. Whether or not conscientiousness is also predictive of success during the clinical phase of medical study is still being debated, and it remains suspenseful to observe how our study participants continue to progress in their studies [10], [18].

Successful students significantly more often articulate a positive motivation for taking up medical studies and justify their desire to study as one that strives to learn and understand more. In contrast, at-risk students express a more subjective dissatisfaction with their former job situation. Comparable results were published in a study by the Medical University of Vienna which describes the “joy of learning” as a predictive factor for success [3]. In addition, the successful students in our cohorts reported being very ambitious and setting personal goals to achieve good grades, whereas the at-risk students demonstrated a certain reservation about making statements about the academic demands they placed on themselves. For instance, study participant S15 stated, *“The grade is not so important, but rather that you take away as much knowledge with you as possible.”* There was, in part, the impression that for these students their current test scores did not bear any relevance to success in future medical practice. Due to the study design, it was not possible to discern if this was indicative of a self-perceived deficiency or if low ambition led to low performance and consequently to uncertain graduation from medical school.

Unlike Heublein et al. [7] have asserted for all students, the financial situation did not pose a reason to discontinue university study in the context of our data. This may be explained by the period of time in which the survey was conducted which was in an early phase of study when financial reserves may not yet be exhausted and additional challenges were not yet apparent, such as applying for an extension of government-funded financial aid (Bafög). Some of the study participants stated that they wanted to work even more during the clinical phase. It would be interesting to see if and to what extent an increase in employment may negatively affect the course of study [7].

Generally, our findings can be applied in two ways. First, the observation that a positive reason for taking up medical studies and strong personal ambition predict success can be included into university selection procedures, possibly even as a survey-based admission procedure if valid criteria can be generated for this [19], [20]. However, it must not be forgotten that the strong desire to be admitted to medical school can distort the test results in terms of the applicants’ social desirability [21]. For this, the development of standardized tests, such as multiple mini-interviews or situational judgment tests, to explore motivation and ambition would be important [22], [23], [24]. Since applicants on the waiting list are admitted by a central administrative body, the *Stiftung für Hochschulzulassung*, individual medical schools do not have the option of selecting the most promising applicants among them. However, our findings are very likely generally applicable to older students with prior professional experience and can be considered in the admission procedures of individual universities.

Second, the differences identified here between successful and at-risk students can serve to recognize early on the risk of prolonged study time or discontinuation and lead to appropriate assistance for affected students. Since we see confirmation of the tendency for problems to occur early in the first semesters as a result of exam failures, the identification of one or more instances of poor course grades could serve as a red flag indicating academic struggle [14]. The university then has the opportunity to intervene in such a way that potential deficits in the study program can be addressed, academic advice and counseling in stress management can be given or, as ultima ratio, the discontinuation of medical study can be jointly considered with the aim of avoiding more years of frustration and failure.

Of great interest here are the questions: which interventions are feasible for the medical schools in terms of the economic aspects and how well are they used by the student body. It is possible that at-risk students have a high reluctance to seek individual academic advising.

Finally, we were also interested in the reasons why students admitted from the waiting list decide to discontinue their medical studies. There was no indication that difficulties financing higher education, inadequate study conditions at universities, poor health, personal or familial reasons or a change of profession played a role in the discontinuation of medical study [7]. Forced disenrollment as a result of failed exams also did not occur. Instead, academic problems stood in the foreground and both study participants who were willing to be interviewed stated that a lack of motivation to prepare to retake exams and a fear of failure were their reasons for discontinuing. Due to the small number of cases we are not able to generalize these findings. Our assumption that, in particular, students with prior professional experience would experience a stronger motivation to learn in practice-based model curricula would be an interesting hypothesis to test out and could be the aim of a future comparative study building on the data collected here.

Based on our observations, positive reasons for taking up medical study, a conscientious personality and strong ambition appear to be the most important resilience factors in successful students who are admitted to medical school from the waiting list. The extent to which these parameters will change given the pending reforms to the medical school admission process and to the medical curriculum itself within the scope of the Master Plan 2020 and the decision of the Cultural Ministers’ Conference is surely worthy of further research [2], [25].

## Acknowledgements

The authors wish to thank Peter Kropp for fruitful discussions and both, the Dean of Studies at the University of Rostock´s Medical School and the Institute of Medical Biochemistry and Molecular Biology for their strong support.

## Funding

This study was funded by the Prorector of the University of Rostock (PSL-UMR-1-16).

## Competing interests

The authors declare that they have no competing interests. 

## Figures and Tables

**Table 1:**
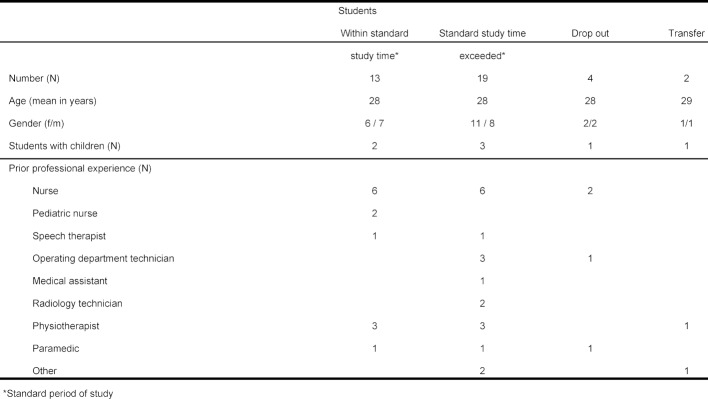
Sociodemographic profile of the study participants
